# STAT3, stem cells, cancer stem cells and p63

**DOI:** 10.1186/s11658-018-0078-0

**Published:** 2018-03-22

**Authors:** Michaela Galoczova, Philip Coates, Borivoj Vojtesek

**Affiliations:** Regional Centre for Applied Molecular Oncology, Masaryk Memorial Cancer Institute, Zluty kopec 7, 656 53 Brno, Czech Republic

**Keywords:** STAT3, Stem cells, Cancer stem cells, p63

## Abstract

Signal Transducer and Activator of Transcription 3 (STAT3) is a transcription factor with many important functions in the biology of normal and transformed cells. Its regulation is highly complex as it is involved in signaling pathways in many different cell types and under a wide variety of conditions. Besides other functions, STAT3 is an important regulator of normal stem cells and cancer stem cells. p63 which is a member of the p53 protein family is also involved in these functions and is both physically and functionally connected with STAT3. This review summarizes STAT3 function and regulation, its role in stem cell and cancer stem cell properties and highlights recent reports about its relationship to p63.

**This article was specially invited by the editors and represents work by leading researchers.**

## Background

Our team is working on expression and functional properties of p63, which is a member of the p53 protein family with diverse roles in carcinogenesis that include tumor-suppressing and oncogenic effects [1–4]. Several recent studies link p63 with STAT3 that is one of the seven members of the Signal Transducer and Activator of Transcription (STAT) family of transcription factors [5]. STAT3 and p63 are important regulators of cell proliferation and survival, and have major roles in the maintenance of stem cells and their differentiation, and are involved in carcinogenesis of many cell types. STAT3 is known to act through its ability to regulate both oncogenes and tumor suppressor genes, as well as influencing tumor microenvironments [6–9]. It exerts a plethora of different and sometimes contrasting functions in normal and transformed cells. This multifaceted function can be partly explained by its involvement in signaling pathways in many different types of cells and conditions [10]. p63 is most commonly linked with epithelial malignancies, particularly squamous cancers [9, 11]. Like STAT3, p63 acts to transcriptionally regulate a wide variety of genes in cancer that are involved in proliferation, survival and differentiation, and also has major roles in cell adhesion and motility [3, 4]. This review will provide basic information about STAT3 and its regulation and will focus on its role(s) in stem cells and cancer stem cells. We will also briefly discuss its relationship with p63 which is also involved in many pathways connected with self-renewal and differentiation properties of stem cells and cancer stem cells [8, 12, 13].

### Structure of STAT3

All STATs share similar functional domains, including an N-terminal domain, a coiled coil domain which enables protein-protein interactions, a central DNA-binding domain, a linker domain that affects DNA-binding stability and a classic SRC homology 2 (SH2) domain. STAT3 has two important phosphorylation sites – a tyrosine residue at amino acid position 705 (Tyr705) within the SH2 domain and a serine phosphorylation site at position 727 (Ser727) within the C-terminal transactivation domain, which is absent in the alternatively spliced STAT3β variant [14].

**Fig. 1:**
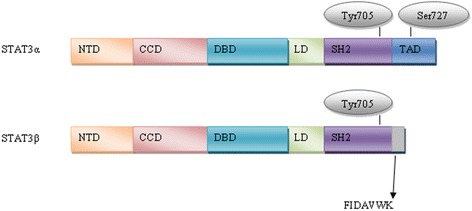
Schematic overview of STAT3α and β isoforms – NTD, NH2-terminal domain; CCD, coiled coil domain; DBD, DNA-binding domain; LD, linker domain; SH2 domain; TAD, transcription activation domain. Figure shows also two key phosphorylation sites, tyrosine 705 and serine 727

The full-length isoform (isoform 1), STAT3α, which is the most commonly expressed form, encodes a protein of predicted mass 88 kDa [15, 16]. The truncated STAT3β isoform (isoform 3) (83 kDa) is produced by alternative splicing of a 3' splice acceptor site in exon 23 of the *STAT3* gene. STAT3α is 770 amino acids in length and STAT3β is identical in sequence with the exception of 55 amino acids at the C-terminal tail that are replaced with a unique seven amino acid sequence (Fig. 1) [15–17]. STAT3β was initially thought to be a negative regulator of STAT3α target genes because it lacks the transactivation domain [15]. However, it was demonstrated *in vivo* that STAT3β is not a dominant negative factor and seems to be involved in lipopolysaccharide-mediated induction of the interleukin-10 promoter [18]. Another two isoforms have been described, produced by limited proteolysis during granulocytic differentiation; a 72 kDa C-terminal-truncated form known as STAT3γ, and a 64 kDa truncated isoform known as STAT3δ [19–21]. Another isoform (isoform 2) was identified with a deleted amino acid at position 701 (Del-Ser701) by global phosphoproteomic approaches [22, 23]. The validity and function of these latter variants remains to be determined.

### Regulation of STAT3

STAT3 activity is regulated by multiple activators and negative regulators, reflecting its varied functions in a wide range of cell types. The main mechanism of activation is phosphorylation of Tyr705 by upstream kinases, although residue Ser727 can also be phosphorylated. Moreover, STAT3 may be transcriptionally active in its unphosphorylated form and its activity is regulated also by other posttranslational modifications such as acetylation, methylation or ubiquitination. Negative regulation of STAT3 is provided by protein phosphatases and specific protein inhibitors – Suppressors of Cytokine Signaling (SOCS) and Protein Inhibitors of Activated STAT (PIAS). Furthermore, its expression is regulated by several miRNAs.

### Activation of STAT3

**Fig. 2:**
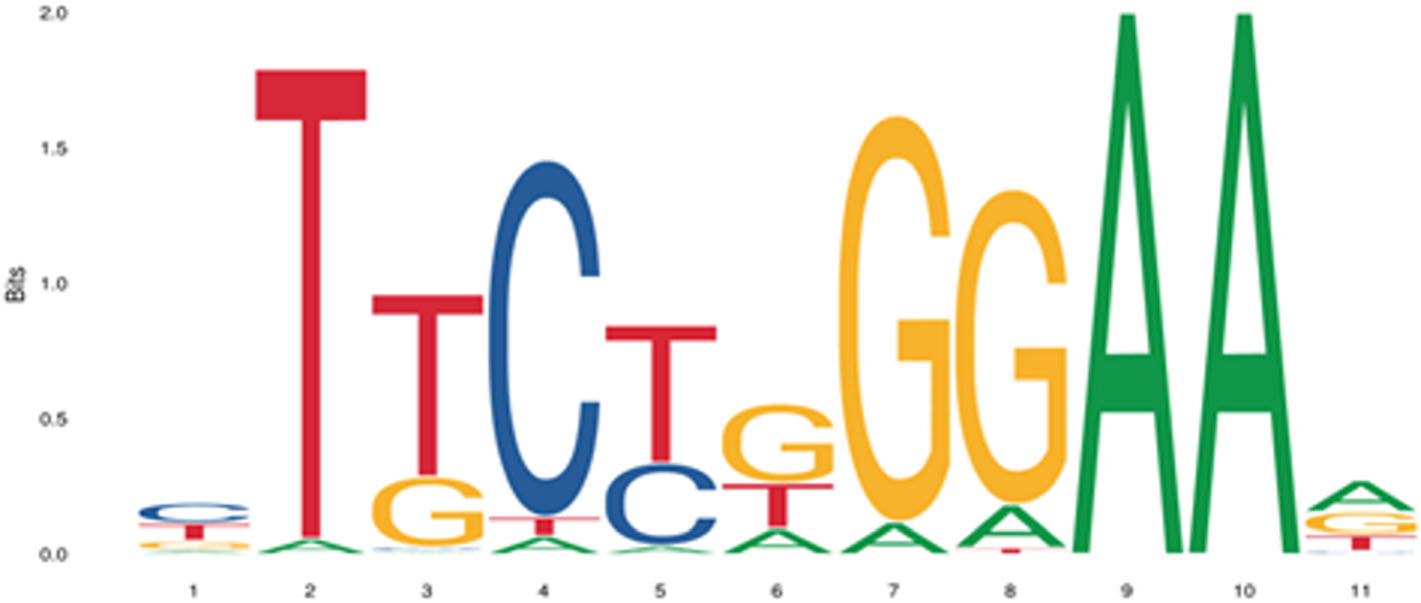
STAT3 consensus binding site from JASPAR database [29]

STAT3 is mainly activated by phosphorylation of the conserved Tyr705 residue, which leads to dimerization by reciprocal phosphotyrosine-SH2 interactions of two monomers [24]. Activated STAT3 dimers translocate to the nucleus through interactions with importins and bind to the GAS (Interferon-γ-Activated Sequence) motif within target gene promoters to activate transcription [25–27]. Most STATs including STAT3 bind to GAS motifs with a consensus TTCN_2-4_GAA [28]. The STAT3 consensus binding site is illustrated in Fig. 2 [29]. Besides STAT3 homodimers, STAT1/STAT3 heterodimers have been reported, with transcriptional potential that differs from STAT1 or STAT3 homodimers [30].

STAT3 Tyr705 phosphorylation is primarily mediated by Janus Kinases (JAKs) associated with cytokine stimulated receptors [31]. The most well-known activator is interleukin 6 (IL-6). However, other members of the IL-6 family are also able to activate STAT3, including IL-10 [32], IL-11 [33], Ciliary Neurotrophic Factor (CNTF) [34], Leukemia Inhibitory Factor (LIF) [35] and Oncostatin [36]. Phosphorylation of Tyr705 is also rapidly increased by receptor tyrosine kinases including Epidermal Growth Factor Receptor (EGFR) [37], Vascular Endothelial Growth Factor Receptor (VEGFR) [38], Platelet-derived Growth Factor Receptor (PDGFR) [39] and Insulin-like Growth Factor 1 Receptor (IGFR) [39, 40] as well as by non-receptor tyrosine kinases like Src-family kinases (Src, Hck, Lyn, Fyn, Fgr) [41], Bcr-Abl [42] and Bone Marrow X-linked non-receptor tyrosine kinase (BMX) [43]. Recent studies also identified Toll-like receptors as Tyr705 activators [44, 45]. Moreover, Tyr705 can be indirectly activated by G-protein coupled receptors such as Sphingosine-1-phosphate Receptor 1 (S1PR1) [46], BV8 [47] or angiotensin II [48]. Engagement of cadherins was also shown to activate STAT3 through up-regulation of IL-6 family cytokines [49].

In addition, STAT3 is phosphorylated at serine 727 (Ser727) by members of the Mitogen-activated Protein Kinases (MAPK) like p38MAPK [50] or Extracellular Signal Regulated Kinases (ERK) [51], by c-Jun N-terminal Kinase families (JNK) [52] and by Protein Kinase C (PKC) [53]. The Mammalian Target of Rapamycin (mTOR) may also phosphorylate STAT3 at Ser727 [54]. It was generally believed that phosphorylation of Tyr705 is necessary for STAT3 activation, whereas Ser727 phosphorylation is required for its maximum activity, presumably by recruiting transcriptional co-factors [55, 56]. However, Ser727 phosphorylation can also reduce p-Tyr705 [57] and recent studies have suggested that STAT3 can be activated through Ser727 phosphorylation in the absence of Tyr705 phosphorylation. For example, a correlation was found between Ser727 phosphorylation in the absence of Tyr705 phosphorylation and survival of neuronal stem cells [58]. Moreover, constitutive activation of Ser727 is essential for the survival of primary human *in vitro* differentiated macrophages [59] and drives prostate carcinogenesis independently of Tyr705 phosphorylation [60]. Above that, STAT3 is constitutively phosphorylated at Ser727 while not at Tyr705 in chronic lymphocytic leukemia [61, 62]. In addition, STAT3 can localize in mitochondria, where is serine phosphorylated and regulates mitochondrial functions independently from its transcriptional activity [63, 64].

Unphosphorylated STAT3 (U-STAT3) may also activate gene transcription. STAT3 nuclear import is independent of tyrosine phosphorylation and is mediated by importin-α3 [65], Ran and importin-beta1 [66]. U-STAT3 dimerization is influenced by disulfide bonds between cysteines [67] and dimers bind to the same GAS DNA-binding site as phosphorylated STAT3 but also bind AT-rich DNA structures to influence chromatin organization [68]. Moreover, U-STAT3 core protein (lacking the N-terminal domain) binds to target *ds*DNA [69]. Several genes (*Cdc2*, *Cyclin B, Mras*, *E2f-1, Rantes)* do not respond directly to phosphorylated STAT3 but are activated in the late phases of IL-6 driven responses when there is an accumulation of U-STAT3 [70]. Some STAT3-responsive genes have kappa B elements, and these genes are activated by a transcription factor complex formed when U-STAT3 binds to unphosphorylated Nuclear Factor Kappa B (NF-κB) [71].

STAT3 dimerization is also positively regulated by reversible acetylation of residue Lys685 by its co-activator p300/CREB-binding protein [72–74] and by tri-methylation of Lys180 [75].

### Negative regulation of STAT3

Dephosphorylation of STAT3 by protein phosphatases plays a major role in regulating STAT3. Multiple protein tyrosine phosphatases such as MEG2 [76], CD45 [77], Src-homology Region 2 Domain-containing Phosphatase 1/2 (SHP1/2) or T-cell Protein Tyrosine Phosphatases (TC-PTP) [78] have been shown to dephosphorylate Tyr705 of STAT3. STAT3 Ser727 can be dephosphorylated by Protein Phosphatase 1 (PP1) [79] or Dual Specificity Protein Phosphatase 2 (DUSP2) [80].

The SOCS proteins negatively regulate JAK/STAT3 signaling through three different mechanisms; inhibition or targeting JAKs for degradation by the proteasome; shielding the STAT3 binding sites on the cytokine receptor; or removing target proteins via ubiquitination and proteasomal degradation [81]. SOCS3 is known to negatively regulate STAT3 activity [82]. PIAS proteins participate in negative regulation during later phases of signaling. They are endogenous inhibitors of STATs that act as E3-type small ubiquitin-like modifier ligases. PIAS3 is known to block the DNA-binding activity of STAT3 and inhibits STAT3-mediated gene activation [83, 84]. Other posttranslational modifications such as methylation of Lys140 can also negatively regulate STAT3 activity [85].

### STAT3 regulation by miRNAs

Several studies have indicated that miRNAs are critical regulators of STAT3. A number of miRNAs have been identified that affect STAT3 signaling in various types of cancers (reviewed in [86]). Moreover, some miRNAs have been shown to play a role in regulating stem cells and cancer stem cell properties. *miR-124* was found to directly target *STAT3* mRNA to regulate cardiomyocyte differentiation of bone marrow-derived mesenchymal stem cells [87]. *miR-1181* inhibits stem cell-like phenotypes and suppresses STAT3 in human pancreatic cancer [88], whilst *miR-7* indirectly inhibits STAT3 and thereby decreases the number of breast cancer stem cells [89].

**Fig. 3:**
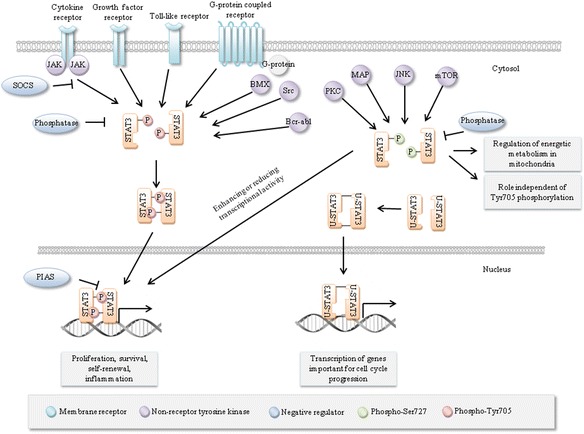
STAT3 regulation. Multiple signals lead to phosphorylation of STAT3 at Tyr705 or Ser727, leading to dimerization, nuclear import and transcriptional activation. SOCS proteins, phosphatases and PIAS proteins inhibit STAT3 activity at different stages of STAT3 activation. Other modifications including ubiquitination, acetylation and methylation may also occur

To summarize the data above, it is evident that STAT3 expression and activation are regulated by multiple signals and they play a role in many signaling pathways. This enables STAT3 to be a flexible and adaptable regulator of cell function in different types of cells under different conditions and regulate gene expression directly or indirectly through other transcription factors [90]. An overview of STAT3 regulation is shown in Fig. 3. This review will now focus on STAT3’s involvement in signaling pathways regulating stem cells and cancer stem cells.

### STAT3 and stem cells

Stem cells are defined by their ability to self-renew and to generate progenitor cells that can subsequently divide and differentiate into the different types of cells of a particular tissue [91]. There are two main types of naturally occurring stem cells: embryonic stem cells, which are isolated from the inner cell mass of blastocysts, and adult stem cells, which are found in various tissues. Embryonic stem cells are considered to be totipotent and can give rise to all cell types in the organism whereas adult stem cells are pluripotent, showing lineage restriction according to the particular tissue in which they reside. The other type of stem cells are induced pluripotent stem cells (iPSCs) that can be generated from adult cells [92].

### Mouse stem cells

#### Mouse embryonic stem cells (mESCs)

LIF and its close relatives (IL-6) family are known to maintain pluripotency of mESCs [93]. The effect of LIF on JAK/STAT3 signaling is mediated through the LIF receptor (LIFR) which consists of two subunits: gp130, which is common for all types of cytokine receptors, and LIF receptor beta (LIFRβ). LIF induces heterodimerization and phosphorylation of these subunits [94] leading to rapid phosphorylation of intracellular non-receptor JAKs that phosphorylate STAT3 on tyrosine residues [95]. This phosphorylation is responsible for STAT3 activation necessary for self-renewal of mESCs. It seems that activated STAT3 is sufficient to maintain the undifferentiated state of mESCs. However, there is a threshold, and mESC lines with lower expression of constitutively active STAT3 are not able to fully inhibit differentiation [96]. STAT3 activation in mESCs leads to expression of genes that are known regulators of pluripotency, such as *Myc* or *Bcl3* [97, 98] and activated STAT3 is known to cooperate with Nanog, another key component of pluripotency [99]. Thus, STAT3 plays a major role in regulating mESCs fate. The regulation is possibly enabled by existence of the two different phosphorylation sites Tyr705 and Ser727, whose modification could switch between self-renewal and differentiation [100].

#### Mouse adult stem cells

Gu et al. [101] demonstrated that suppression of STAT3 promotes neurogenesis and inhibits astrogliogenesis in neural stem cells. Moreover, Kamakura et al. [102] showed a crosstalk between differentiation pathways where STAT3 is activated in the presence of active Notch as well as the Notch effectors Hes1 and Hes5. However, STAT3 plays a role not only during postnatal development of the mouse neocortex, the interaction between the JAK/STAT3 and Notch ligand Delta-like1-Notch signaling pathways plays an essential role in maintaining neural precursors during early neocortical development [103]. STAT3 also seems to be an important regulator of hematopoietic regeneration [104], self-renewal of adult muscle satellite cells during injury-induced muscle regeneration [105], regeneration of airway ciliated cells from basal stem cells [106], adipogenesis [107], differentiation of multiciliated [106] and hair cells [108] in mouse models. Furthermore, STAT3 is required to maintain the full differentiation potential of mouse mammary stem cells and the proliferative potential of mammary luminal progenitors [109].

### Human stem cells

#### Human embryonic stem cells (hESCs)

As mentioned above, stemmness of mES cells is maintained through a signaling pathway including the IL-6 family of cytokines, JAKs and STAT3. However, this pathway has little effect on hESCs [93]. Human LIF can induce STAT3 phosphorylation and nuclear transportation through gp130/LIFRβ, but is unable to maintain the pluripotent state of hESCs [110]. However, Yang et al. [111] showed that increased STAT3 activation is sufficient to convert epiblast-derived stem cells to naive pluripotency and Chen et al. [112] observed that temporarily increasing STAT3 activity is sufficient to reprogram hESCs to naive-like pluripotent cells. Hence, STAT3 activation is a limiting factor in somatic cell reprogramming.

#### Human adult stem cells

There are numerous studies that have investigated the role of STAT3 in human mesenchymal stem cells (hMSCs). hMSCs are a heterogeneous population of non-hematopoietic precursor cells predominantly found in the bone marrow. Matsui et al. [113] demonstrated that hMSCs protect against obstruction-induced renal fibrosis by decreasing STAT3 activation and STAT3-dependent Matrix Metallopeptidase 9 production. Interesting to note is the interaction of hMSCs with cancer cells via cytokine networks. Hsu et al. [114] showed that the IL-6/JAK/STAT3 pathway could be activated by hMSCs when they are co-cultured with lung cancer cells to enhance lung cancer initiation. Rattigan et al. [115] illustrated that IL-6, which is produced and secreted at high levels by breast cancer cells in response to hypoxia, regulates hMSC migration towards cancer cells. IL-6 binds to its receptor on hMSCs, leading to STAT3 activation to promote hMSCs migration and survival.

### STAT3, cancer and cancer stem cells

**Fig. 4:**
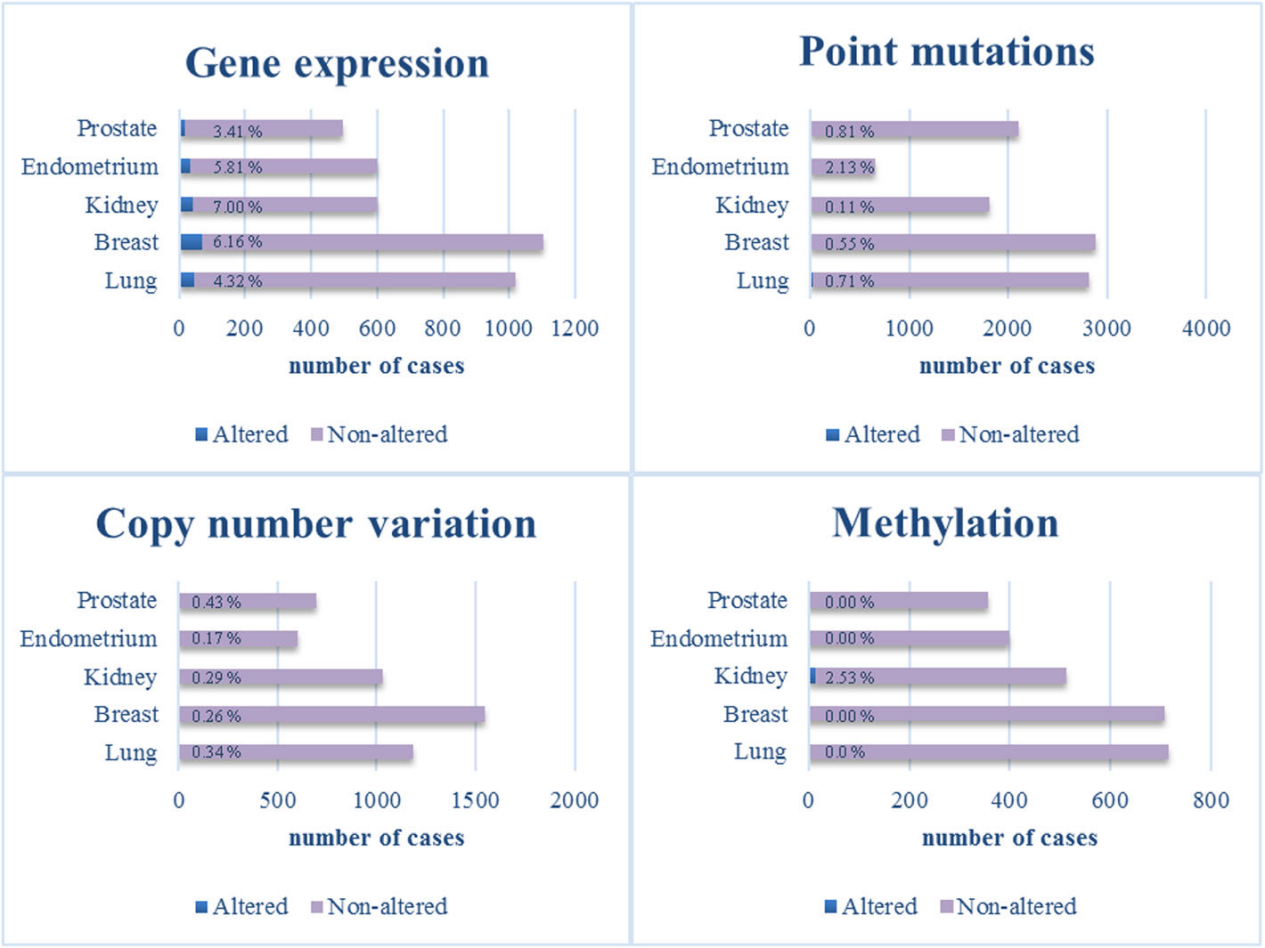
STAT3 mutational status. The table showing the distribution of mutations across the primary tissue types that are curated by COSMIC database was used as template. Only cancer types with all mutation types included were chosen for this picture [118]

STAT3 is constitutively activated in many types of human solid tumors and hematological malignancies [116]. For example, STAT3 activation occurs in more than 40% of breast cancers, most often in the triple negative subtype that lack estrogen receptor, progesterone receptor and Human Epidermal Growth Factor 2 (HER2) amplification [5, 117]. However, the *STAT3* gene is very rarely altered in human malignancies by copy number variation, point mutation or methylation and rarely by gene expression according to the Catalogue of Somatic Mutations in Cancer (COSMIC) database (Fig. 4) [118].

**Fig. 5:**
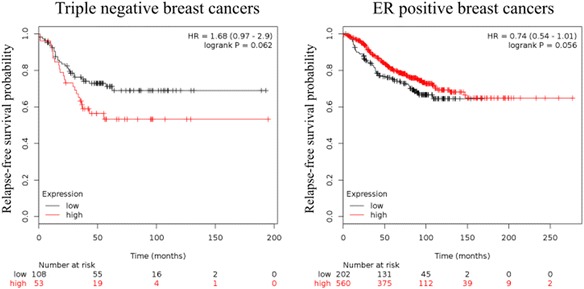
Kaplan-Meier plot of *STAT3* mRNA levels in triple negative and ER^+^ breast cancers. Affymetrix ID for *STAT3* was 225289_at. Relapse-free survival of patients was analysed. JetSet best probe set and auto select best cut-off were used for analysis [122]

In the absence of genetic alterations, constitutive activation occurs through upstream factors such as growth factor or cytokine production acting through paracrine or autocrine pathways; amplification or activating mutations in related receptors; mutations in kinase signaling cascade pathway genes; and/or the loss of negative regulators of STAT3 activity. Constitutive activation of STAT3 is predictive of poor prognosis in many types of cancer [119–121] and although STAT3 is only rarely altered by gene expression changes, mRNA levels show a similar trend. Using publicly available array profiling data, we could show here that in triple negative breast cancer, which is a representative cancer with constitutive STAT3 activation, higher mRNA levels show a trend for worse relapse-free survival (RFS). Conversely, in ER^+^ breast cancer where the activation of STAT3 is low, high *STAT3* mRNA levels indicate a better RFS probability (Fig. 5) [122].

There is an opposite trend also between the main groups in ovarian and lung cancer. Low *STAT3* mRNA levels indicate better progression-free survival (PFS) in serous ovarian cancer, whereas high levels point to better PFS in endometrioid ovarian cancer. Low *STAT3* mRNA levels in lung adenocarcinoma and oppositely high levels in squamous cell lung cancer indicate better first progression (FP) probability. In gastric cancer, high *STAT3* mRNA levels indicate worse FP probability. For these analyses we used KM-plotter cited in [123] (Table 1).

**Table 1:** Patient outcomes in relation to *STAT3* mRNA expression in breast, ovarian, lung and gastric cancers

Breast cancer	All types (*n*=1764)	Triple negative (*n*=161)	ER positive (*n*=762)
RFS probability	0.0530 ↓	0.0620 ↓	0.0560 ↑
Ovarian cancer	All types (*n*=614)	Serous (*n*=483)	Endometrioid (*n*=44)
PFS probability	0.0160 ↓	0.0150 ↓	0.0720 ↑
Lung cancer	All types (*n*=596)	Adenocarcinoma (*n*=443)	Squamous cell (*n*=141)
FP probability	0.020 ↓	0.0058 ↓	0.0002 ↑
Gastric cancer	All types (*n*=522)		
FP probability	0.3300 ↑		

Patient outcomes in relation to *STAT3* mRNA expression are described by logrank *p* value. Affymetrix ID for *STAT3* was 225289_at. Relapse-free survival (RFS) was analysed in breast cancer, progression-free survival (PFS) in ovarian cancer and first-progression (FP) in lung and gastric cancers. JetSet best probe set and auto select best cut-off were used for analysis from Kaplan-Meier plot [123]. Upward arrows indicate that higher *STAT3* levels correlate with improved survival; downward arrows indicate that lower *STAT3* levels associate with improved survival.

As mentioned at the beginning, STAT3 is also important in tumor biology for its ability to promote cancer through regulating cancer stem cell activities. It is widely accepted that tumors contain a sub-population of cells that share properties with normal tissue stem cells, called cancer stem cells or cancer stem-like cells (CSCs) [124–128]. However, the CSC theory remains controversial because of the variety of differences between CSCs and normal stem cells. As stem cells, CSCs have the ability to self-renew, however while normal stem cells are able to differentiate into multiple distinct cell types, most CSCs differentiate into only a single cell type, the cells which form the bulk of the tumor. However, an evidence for multilineage differentiation potential of CSCs was reported in colon carcinomas and leukemia. Another difference is that while the phenotypes of normal stem cells seem to be fixed, the phenotypes of CSCs vary from one tumor to another tumor of the same molecular/pathological type, most likely because they are affected by the abnormalities resulting from the process of neoplastic transformation [127]. CSC identification and understanding of their biology could have critical clinical relevance, because CSCs are uniquely able to reform the tumor and exhibit enhanced resistance to cancer treatments [124–128]. Notably, as mentioned above, STAT3 is often constitutively activated in triple negative breast cancers and these cancers display a profile of cell surface markers that is similar to that of breast CSCs [117, 126, 129]. Below, we summarize the evidence for the role of STAT3 in CSCs properties in the common human malignancies.

STAT3 was reported to have an essential role in maintaining the expression of genes that are important for stem cell phenotype and are used as markers of CSCs. Many putative CSC markers have been identified, the most commonly used are expression of transmembrane glycoproteins CD24, CD34, CD38, CD44, CD90 and CD133, together with Aldehyde Dehydrogenase (ALDH), the ability to form spheroids in suspension *in vitro* and the ability to exclude cell permeable dyes such as Hoechst33342 for side population assessment [127]. The STAT3 pathway is preferentially active in subpopulations of cells enriched for CSC markers and its inhibition decreases cell viability and tumorsphere formation. On the other hand, several proteins that stimulate cell growth and proliferation reduce its activity [117, 130, 131]. Importantly, STAT3 can form a complex with internalized CD44 and acetyltransferase p300, inducing STAT3 acetylation at Lys685, dimer formation and translocation to the nucleus where it binds to the promoters of genes including cell cycle regulators *cyclin D1* [47] or *Myc* and *Twist1* [132]. Furthermore, STAT3 was found to physically interact with CD44 and NF-κB and activate the catalytic subunit of telomerase to prolong proliferative potential [133]. Moreover, activated STAT3 can increase CD133 expression through functional cooperation with NF-κB and Hypoxia Inducible Factor 1 Alpha (HIF-1α) [134].

Nowadays, epithelial-mesenchymal transition (EMT) and tumor microenvironments are highly discussed topics in the context of CSCs. There is increasing evidence pointing to plasticity between CSCs and their more differentiated derivatives. It is considered that whereas CSCs can differentiate into non-CSCs, the reverse process is also possible via EMT, which is a transdifferentiation program required for tissue morphogenesis during embryonic development [135, 136]. Over and above that, several studies reported direct links between EMT and gain of CSC properties [137, 138]. EMT and CSC formation is a dynamic process triggered by multiple shared signaling pathways, such as Transforming Growth Factor β (TGF-β), Wnt/β-catenin, Hedgehog, Notch, NF-κB and others [139, 140].

It was reported that hepatocellular carcinoma could arise from IL-6/STAT3 driven transformed stem cells with inactivated TGF-β signaling and that human hepatocellular cancer cells expressing STAT3 and the putative stem cell markers Octamer-binding Transcription Factor 4 (OCT4) and Nanog lost pro-differentiation proteins TGF-β-receptor type II and Embryonic Liver Fodrin [141]. In addition to this role in hepatocellular cancer, STAT3 activation plays a role in EMT induction in different types of tumors. STAT3 can be activated by IL-6 dependent or independent mechanisms such as a non-canonical Frizzled 2 pathway [142] or TGF-β/LIF [143]. Moreover, induction of EMT after STAT3 activation and expansion of the CSC population were observed in relation to resistance to cisplatin or trastuzumab [144, 145]. The mechanisms of trastuzumab resistance, which is a HER2-targeting antibody used to treat HER2^+^ breast cancer, are well-documented. Inactivation of Phosphatase and Tensin Homolog (PTEN) leads to increased resistance to this drug and it seems that STAT3 is a negative regulator of PTEN among trastuzumab-resistant cells [146, 147]. An IL-6 loop was found, where IL-6 activates the AKT, STAT3 and NF-κB pathways while suppressing PTEN expression [146]. Recently, it was found that trastuzumab resistance in this type of cancer is promoted through activation of a STAT3/HIF-1α/Hes1 axis via down-regulation of PTEN [147].

STAT3 activation was observed also in HER2 negative breast cancer, where activation of STAT3 also correlates with CSC properties. The STAT3 pathway is positively regulated by mTOR signaling in this context, whereas PTEN serves as a negative regulator of both STAT3 and mTOR [148]. Moreover, PTEN appears to function as a crucial inhibitor of glioblastoma stem cells through mediating cooperative perturbation of AKT and STAT3 signals [149].

Furthermore, STAT3 is a critical transcription factor in angiogenesis; it participates in expression and protein stability of HIF-1α and regulates or is itself regulated by VEGF. This involvement was also shown to play a role in maintaining the self-renewal properties of CSCs [38]. Interestingly, VEGF-mediated angiogenesis was reported to link EMT-induced cancer stemness to tumor initiation [150].

STAT3 is also involved in the regulation of NF-κB signaling in tumor cells and in non-transformed stromal cells in the tumor microenvironment. STAT3 physically interacts and functionally cooperates with NF-κB in tumor cells and also in tumor-associated immune cells [7, 47, 134]. Among tumor-associated immune cells, tumor-associated macrophages were found to promote CSC-like phenotypes through Milk Fat Globule-EGF Factor 8 (MGF-E8)/STAT3 and Sonic Hedgehog pathways, or through EGFR/STAT3/Sox2 [151, 152]. Moreover, it was also shown that mast cells modulate proliferation, migration and stemness through down-regulation of GSK3β and inhibition of STAT3 activation [153].

STAT3 can be activated and thereby contribute to CSCs properties by the BMX [43] and Ras homolog family member C [154] and it can be activated also epigenetically by the histone-lysine N-methyltransferase Enhancer of Zeste Homolog 2 (EZH2) [75].

Taking the above observations together, the roles STAT3 in promoting and maintaining CSC properties are highly complex. STAT3 directly interacts with transmembrane glycoproteins which are expressed by normal stem cells and are widely used as markers to identify and isolate CSCs. STAT3 is involved in pathways that are connected with EMT, which is one of the major proposed mechanisms of generating CSCs. Moreover, it plays a critical role in angiogenesis and participates in regulating the tumor microenvironment that provides signals for differentiation or proliferation especially through its involvement in inflammatory NF-κB pathway. In addition, feedback activation of STAT3 may play a prominent role in mediating drug resistance to a broad spectrum of targeted cancer therapies and chemotherapies [155]. Although it seems to be an ideal target for anti-cancer therapy, effective approaches to inhibit STAT3 are still missing. This lack is caused presumably because of the complexity of STAT3’s biology in normal as well as cancer cells and also because it lacks enzymatic activity, making it a challenging target [86]. Inhibitors of STAT3 which are currently tested are reviewed in [155], although no inhibitor that directly targets STAT3 has yet been approved by the US Food and Drug Administration for clinical use. However, several tyrosine kinase inhibitors are in the clinic such as sorafenib and sunitinib that can inhibit STAT3 signaling indirectly, leading to tumor cell cycle arrest and apoptosis [156, 157].

As was mentioned in the beginning, there is increasing evidence that STAT3 activation and p63 expression are connected; hence their relationship will be briefly discussed in the following part of this review.

### The connections between STAT3 and p63

**Fig. 6:**
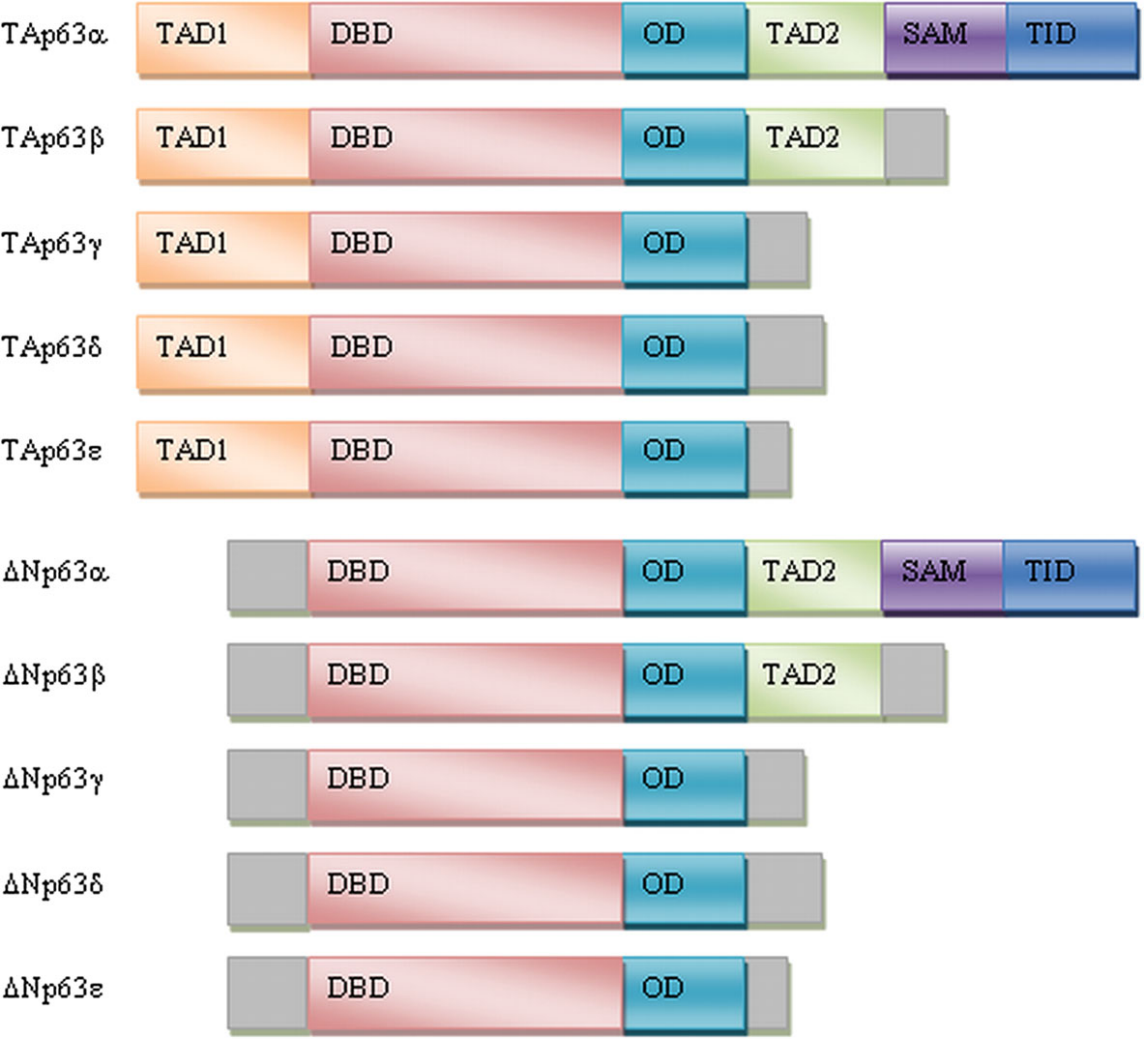
Schematic overview of p63 isoforms **–** TAD1, transcription activation domain 1; DBD, DNA-binding domain; OD, oligomerization domain; TAD2, transcription activation domain 2; SAM, sterile α-motif; TID, transcription inhibitory domain

p63 is a member of the p53 family of transcription factors that consists of p53, p63 and p73 proteins. All family members have important functions in tumorigenesis and morphogenesis and share the same domain organization including an N-terminal transcription activation domain (TAD), a DNA binding domain (DBD) and a C-terminal oligomerization domain (OD). They act as tetramers and due to their partial homology in the oligomerization domain they may form heterotetramers. They also have highly homologous DNA binding domains, indicating that they are able to bind to the other family members target genes. The *TP63* gene is localized on chromosome 3 and gives rise to multiple isoforms due to differential promoter selection (full-length TA and N-terminal truncated ΔNp63) and alternative splicing of the 3´end of the mRNA (α, β, γ, δ, ε) [158] (Fig. 6). ΔNp63 isoforms lack the N-terminal transactivation domain, hence they are able to antagonize full-length isoforms of p63 and also other p53 family members and act like dominant negative transcription inhibitors. Nevertheless, they also have transactivation activity due to the presence of an alternative TAD [158, 159]. Among C-terminal isoforms, p63α isoforms have a sterile alpha motif (SAM) that is known to be involved in protein-protein interactions and they have a transcription inhibitory domain (TID), which inhibits its transcriptional activity [160, 161].

p63 is well known for its role in epidermal development. The importance of this protein during this process was established using p63 null mice. Those mice showed a complete lack of stratified squamous epithelia and their derivatives or they had stratified but disrupted epidermis depending on the used model. They also had absent or truncated limbs and craniofacial abnormalities [162, 163]. The predominant isoform in epithelial tissues is ΔNp63α which is highly expressed in the basal cells of stratified and glandular epithelia, including epidermis, and its levels decrease with cellular differentiation. Oppositely, TAp63 positive cells are located suprabasally in stratified epithelia, indicating a switch between isoforms during differentiation [1, 2, 9]. ΔNp63 protein expression is restricted to other basal cells including those in breast, prostate, bladder and colorectum. Thus, it is widely used as a marker for this type of cells [1, 11, 12, 164]. Interestingly, activated STAT3 plays a role in promoting the regeneration of airway ciliated cells from basal stem cells [106] and is also involved in malignant transformation of foregut basal progenitor cells [165]. Furthermore, it was shown using ChIP-Seq analysis that p63 co-operates with STAT3 in human keratinocytes [166].

p63’s role in tumorigenesis is complex, also because it seems that TAp63 and ΔNp63 isoforms play opposite roles in this process. Like *STAT3*, *TP63* is rarely mutated in human cancer, but p63 activity is often increased. One mechanism for increased activity of p63 is gene amplification, and many tumors with amplification show increased p63 expression [9]. ΔNp63 is supposed to behave as oncoprotein and is up-regulated in squamous cell carcinomas [11, 167] and triple negative basal-like breast tumors [4] amongst other tumor types. It also plays roles in a variety of pathways that are implicated in CSC properties, reviewed in [8]. In addition, ΔNp63 increases the expression of Wnt receptor Frizzled 7 thereby enhancing Wnt signaling which leads to promotion of normal mammary stem cell activity and tumor initiating activity in the basal-like subtype of breast cancer [168]. Further, Memmi et al. [13] showed a positive modulation of Hedgehog signaling pathway by ΔNp63 to maintain self-renewal potential of mammary CSCs. On the other hand, TAp63 shares the abilities of the “guardian of the genome” p53 to induce cell cycle arrest and apoptosis and TAp63 may thus act as tumor suppressor. However, there are also reports that it could behave as oncogene. For example, TAp63 is the predominant isoform expressed in hematological malignancies, and it was shown that TAp63 over-expression leads to increased tumor progression of head and neck squamous cell carcinoma. It is also expressed in colon carcinoma [1, 169].

STAT3 is often given in connection with ΔNp63. Both ΔNp63 [3, 4, 13, 129] and STAT3 [117, 170, 171] were suggested as CSC markers and are associated with triple negative breast tumors that show more CSC markers than non-triple negative. STAT3 is also frequently constitutively activated in squamous cell carcinomas [121, 172], where ΔNp63 is often over-expressed. Furthermore, they were both reported as master regulators of mammary cancer stem cell maintenance [13, 173]. Additionally, the dual-regulatory effect ΔNp63 on its own promoter is dependent on STAT3 activation and it was confirmed that STAT3 binds to the ΔNp63 promoter [159, 174] and regulates proliferation and differentiation of rabbit limbal epithelial cells via a ΔNp63 mechanisms [175]. There is a possible mechanism involving EGFR signaling pathway that could regulate STAT3 and ΔNp63 activation and expression. It was observed that ΔNp63 expression is regulated by EGFR/STAT3 axis and this is crucial for proliferation of CSCs [176]. Oppositely, we found that ΔNp63 activates EGFR signaling in triple negative breast cancer [4]. Moreover, STAT3 is activated by mTOR and thereby p63 expression is induced and in turn activates Notch signaling through stimulation of *Jag1* gene expression and impedes murine and human cell differentiation [177]. Besides the above-mentioned evidence that STAT3 and ΔNp63 are closely linked, ΔNp63 is also involved in inflammatory NF-κB pathway [178, 179], angiogenesis through VEGF [180], and EMT. There are numerous papers about ΔNp63 involvement in signaling pathways connected with EMT (reviewed in [181]). Some papers claim that ΔNp63 promotes EMT and reduces the opposite process of mesenchymal-epithelial transition (MET), whereas others provide evidence for an opposite role of ΔNp63. To help with explaining contradictory reports could serve a paper [182] where they describe that p63 can trigger the Notch signaling pathway in neighboring cells to potentially promote EMT. Interestingly, Su et al. [183] recently showed that TAp63 is crucial for the transition of mammary cancer cells to acquire characteristic of tumor-initiating cells.

Studying the relationships of p63 with STAT3 and generally the role of p63 in cancer needs careful assessment of isoforms that are being expressed. Moreover, p63 isoforms must be studied in close relationship with the other p53 family members because of the existence of the many possible interactions between p53, p63 and p73 [8].

## Conclusion

STAT3 signaling is a major regulatory pathway of mouse embryonic stem cell fate and also a limiting factor in human somatic cell reprogramming and plays important roles in the maintenance and proliferation of adult stem cells. STAT3 activation occurs during many aspects of carcinogenesis, including involvement in regulating CSC properties. Together with ΔNp63 it was suggested as a marker of CSCs, a major regulator of mammary CSCs maintenance and both are mostly associated with triple negative tumors. They were found to directly interact and they are both involved in several common pathways regulating CSC properties, however their relationship is still not well established and remains to be determined.

## Abbreviations

ALDH: Aldehyde Dehydrogenase
BMX: Bone Marrow X-linked non-receptor tyrosine kinase
CCD: Coiled Coil Domain
CNTF: Ciliary Neurotrophic Factor
COSMIC: Catalogue of Somatic Mutations in Cancer
CSCs: Cancer Stem Cells
DBD: DNA-binding Domain
DUSP2: Dual Specificity Protein Phosphatase 2
EGFR: Epidermal Growth Factor Receptor
EMT: Epithelial-mesenchymal Transition
ER: Estrogen Receptor
ERK: Extracellular Signal Regulated Kinase
EZH2: Enhancer of Zeste Homolog 2
FP: First Progression
GAS: Interferon-Gamma-Activated Sequence
HER2: Human Epidermal Growth Factor Receptor 2
hESCs: Human Embryonic Stem Cells
HIF-1α: Hypoxia Inducible Factor Alpha
hMSCS: Human Mesenchymal Stem Cells
IGFR: Insulin-like Growth Factor 1 Receptor
IL: Interleukin
iPSCs: Induced Pluripotent Stem Cells
JAK: Janus Kinase
JNK: c-Jun N-terminal Kinase
LD: Linker Domain
LIF: Leukemia Inhibitory Factor
LIFR: Leukemia Inhibitory Factor Receptor
LIFRβ: Leukemia Inhibitory Factor Receptor Beta
MAPK: Mitogen Activated Protein Kinase
mESCs: Mouse Embryonic Stem Cells
MET: Mesenchymal-epithelial Transition
MGF-E8: Milk Fat Globule-EGF Factor 8
mTOR: Mammalian Target of Rapamycin
NF-κB: Nuclear Factor Kappa B
NTD: NH2-terminal Domain
OCT4: Octamer-binding Transcription Factor 4
OD: Oligomerization Domain
PDGFR: Platelet-derived Growth Factor Receptor
PFS: Progression-free Survival
PIAS: Protein Inhibitors of Activated STAT
PKC: Protein Kinase C
PP1: Protein Phosphatase 1
PTEN: Phosphatase and Tensin Homolog
RFS: Relapse-free Survival
S1PR1: Sphingosine-1-phosphate Receptor 1
SAM: Sterile Alpha Motif
SH2: Src-homology 2
SHP1/2: Src-homology Region 2 Domain-containing Phosphatase 1 and Src-homology Region 2 Domain-containing Phosphatase 2
SOCS: Suppressors of Cytokine Signaling
STAT: Signal Transducer and Activator of Transcription
TAD: Transcription Activation Domain
TC-PTP: T-cell Protein Tyrosine Phosphatase
TGF-β: Transforming Growth Factor Beta
TID: Transcription Inhibitory Domain
U-STAT3: Unphosphorylated STAT3
VEGFR: Vascular Endothelial Growth Factor Receptor
